# Cerebral Microbleeds With Atrial Fibrillation After Ablation Therapy

**DOI:** 10.3389/fncel.2022.818288

**Published:** 2022-02-14

**Authors:** Yoshinori Hirata, Natsuko Kato, Kanako Muraga, Akihiro Shindo, Naoko Nakamura, Keita Matsuura, Yuichiro Ii, Mariko Shiga, Ken-ichi Tabei, Masayuki Satoh, Tomoyuki Fukuma, Yoshihiko Kagawa, Satoshi Fujita, Ryota Kogue, Maki Umino, Masayuki Maeda, Hajime Sakuma, Kaoru Dohi, Hidekazu Tomimoto

**Affiliations:** ^1^Department of Neurology, Mie University Graduate School of Medicine, Tsu, Japan; ^2^Department of Dementia Prevention and Therapeutics, Mie University Graduate School of Medicine, Tsu, Japan; ^3^Department of Neurology, Nippon Medical School Musashi Kosugi Hospital, Kanagawa, Japan; ^4^Department of Cardiology and Nephrology, Mie University Graduate School of Medicine, Tsu, Japan; ^5^Department of Radiology, Mie University Graduate School of Medicine, Tsu, Japan; ^6^Department of Neuroradiology, Mie University Graduate School of Medicine, Tsu, Japan

**Keywords:** cerebral microbleeds (CMBs), atrial fibrillation (AF), cerebral infarction, small vessel disease (SVD), cognitive impairment

## Abstract

**Background:**

The prevalence of cerebral microbleeds (CMBs) is significantly higher in patients with atrial fibrillation (AF) than in those without AF. CMBs in patients with AF have been reported to be primarily of the lobar type, but the exact cause of this remains unknown. We investigated the possibility that hemorrhagic transformation of embolic microinfarction can account for *de novo* lobar CMBs.

**Methods:**

A total of 101 patients who underwent ablation therapy for AF were prospectively registered, and 72 patients completed the assessment with MRI 6 months after catheter ablation. Brain MRI, including diffusion-weighted imaging (DWI) and susceptibility-weighted imaging (SWI), were examined at 1–3 days (baseline) and 6 months after catheter ablation. We quantitatively evaluated the spatial and temporal distribution of embolic microinfarctions and *de novo* CMBs.

**Results:**

Of the 101 patients, 68 were enrolled in this study. Fifty-nine patients (86.8%) showed embolic microinfarctions on baseline DWI immediately after catheter ablation. There were 137 CMBs in SWI, and 96 CMBs were of the lobar type. Six months later, there were 208 CMBs, including 71 *de novo* CMBs, and 60 of 71 (84.5%) were of the lobar type. Of the 71 *de novo* CMBs, 56 (78.9%) corresponded to the location of previous embolic microinfarctions found on baseline DWI. The platelet count was significantly lower and hematocrit/hemoglobin and Fazekas score were higher in the group with *de novo* CMBs than in the group without *de novo* CMBs.

**Conclusion:**

*De novo* CMBs frequently appeared after catheter ablation therapy. Our results suggest that embolic microinfarction can cause lobar CMBs.

## Introduction

Cerebral microbleeds (CMBs) are small perivascular accumulations of hemosiderin-containing macrophages as a result of extravasation of erythrocytes from cerebral small vessels on histopathological examinations. In neuroimaging, CMBs are defined as small hypointense foci < 10 mm in diameter on magnetic resonance imaging (MRI) using T2*-weighted gradient-recalled echo or susceptibility-weighted imaging (SWI) (Greenberg et al., 2009; Matsuyama et al., 2017; Ogawa Ito et al., 2019). CMBs are classified into two types according to their location (deep and lobar CMBs), and histopathological analysis reveals mainly two types of vascular pathological changes, hypertensive vasculopathy and cerebral amyloid angiopathy (CAA), respectively. Strictly, the lobar CMBs are mostly considered to be caused by CAA and are found frequently in patients with Alzheimer’s disease (Yates et al., 2014; Selim and Diener, 2017), whereas the non-lobar, deep or infratentorial, and mixed types of CMBs are considered to be due to hypertensive vasculopathy (Matsuyama et al., 2017).

Atrial fibrillation (AF) is a common arrhythmia, and its prevalence is increasing worldwide (Bunch, 2020). AF is associated not only with ischemic stroke, but also dementia. AF and dementia share multiple risk factors, and a meta-analysis study has revealed that there is an increased risk of dementia either with or without stroke (Kwok et al., 2011). One plausible cause of dementia with AF is the presence of cerebral infarctions (Graff-Radford et al., 2016) and the other mechanism may be the existence of CMBs which have been suggested to be associated with cognitive dysfunction (Poels et al., 2012).

The prevalence of CMBs is significantly higher in patients with AF than in those without AF (Saito et al., 2014; Horstmann et al., 2015; Selim and Diener, 2017), and CMBs in patients with AF have been reported to be primarily of the lobar type (Vernooij et al., 2009; Selim and Diener, 2017). The underlying pathophysiology of strictly lobar CMBs in patients with AF is yet to be ascertained (Selim and Diener, 2017). In a previous study, we have reported alleviation of cognitive dysfunction after performing catheter ablation in patients with AF (Kato et al., 2021), and observed *de novo* appearance of CMBs in correspondence with preexisting embolic microinfarctions. To investigate the possibility that hemorrhagic transformation of embolic microinfarction can account for *de novo* CMBs in patients with AF, we quantitatively investigated the association between embolic microinfarctions and CMBs on brain MRI.

## Patients and Methods

### Study Protocol

This prospective study was approved by the ethical review board of Mie University Hospital (certificate number 3,038), and all patients provided written informed consent. All patients were recruited from the Department of Cardiology, Mie University Hospital between August 2017 and September 2018. We recruited 101 patients who were admitted to the hospital for AF catheter ablation (Kato et al., 2021).

We obtained clinical information, laboratory, and imaging data at baseline and follow-up after catheter ablation. Detailed clinical information, including age; sex; height; weight; blood pressure; heart rate; medical history, such as hypertension, hyperlipidemia, and diabetes mellitus; history of transient ischemic attack and/or stroke; and medication at baseline, was collected.

### Ablation Procedure

Catheter ablation was performed as described previously (Sutter et al., 2020). After obtaining informed consent, an electrophysiological study was performed in the post-absorptive state under light sedation. After internal jugular and femoral vein punctures, a heparin bolus (100 U/kg) was administered, and continuous infusion of heparin was provided thereafter to maintain an activated clotting time between 250 and 350 s. A diagnostic duodecapolar catheter was placed in the coronary sinus *via* the jugular vein. Three long sheaths were inserted through the femoral vein and introduced into the left atrium (LA) through a single transseptal puncture guided by intracardiac echocardiography. An eicosapolar circumferential catheter (Lasso 2515, Biosense Webster, Diamond Bar, CA, United States) and a multispline mapping catheter (PentaRay, Biosense Webster) were introduced into the LA through the transseptal long sheaths.

All imaging was performed using a biplane flat panel detector angiographic suite (Allura Xper FD10/10 Angio system; Philips Healthcare, Best, the Netherlands). Electroanatomical mapping was performed using the CARTO3 mapping system (Biosense Webster). Radiofrequency ablation was performed with an irrigated catheter (EZ Steer Thermocool, Biosense Webster) using 0.9% normal saline and a point-by-point technique. Extensive encircling pulmonary vein isolation (EEPVI) was performed in patients with paroxysmal AF, and entrance and exit blocks were documented in all patients using Lasso2515 and PentaRay multipolar catheters. In addition to EEPVI, patients with persistent AF received LA posterior wall isolation. Additional linear ablation was performed along the LA roof to connect the left superior pulmonary vein to the right superior pulmonary vein and linear ablation along the LA floor to connect the inferior margin of the left inferior pulmonary vein to the right inferior pulmonary vein to obtain a block into the posterior wall. A bidirectional block was confirmed across all linear ablations using differential pacing techniques. If common atrial flutter was induced by atrial tachycardia pacing, cavotricuspid isthmus line ablation was performed in patients with paroxysmal AF and persistent AF.

### Magnetic Resonance Imaging Protocol

MRI studies were performed at 1–3 days (baseline) and 6 months after ablation (follow-up) with a 3T MR unit (Ingenia, Philips Medical System, The Netherlands) using a 32-channel phased-array head coil (Kato et al., 2021). We used diffusion-weighted imaging (DWI), three-dimensional (3D) fluid-attenuated inversion recovery (3D-FLAIR), 3D double inversion recovery (3D-DIR), and 3D T1-weighted imaging (3D-T1WI) to detect microemboli (Ii et al., 2013, 2019). Acute microinfarctions were diagnosed using DWI images, whereas chronic microinfarctions were evaluated using 3D-DIR, 3D-FLAIR, and 3D-T1WI images. SWI was used to detect CMBs.

The parameters of the 3D-DIR were as follows: field of view, 250 mm; matrix, 208 × 163 (256 × 256 after reconstruction); in-plane resolution, 0.98 mm × 0.98 mm); section thickness, 1 mm with overcontiguous slice; turbo spin echo (TSE) factor 173; repetition time (ms)/echo time (ms), 5,500/247; long inversion time (ms)/short inversion time (ms), 2,550/450; number of signals acquired, two; and acquisition time, 5 min and 13 s.

The parameters of 3D-FLAIR were as follows: field of view, 250 mm; matrix, 256 × 184 (480 × 480 after reconstruction); in-plane resolution, 0.52 × 0.52 mm; slice thickness, 1 mm with overcontiguous slice; TSE factor 203; repetition time (ms)/echo time (ms), 6,000/390; inversion time, 2,000 ms; number of signals acquired, two; and acquisition time, 4 min and 42 s.

3D-T1WI images used turbo-field echo sequences, with the following parameters: field of view, 260 mm; matrix, 288 × 288 (384 × 384 after reconstruction); in-plane resolution, 0.68 × 0.68 mm; slice thickness, 1 mm; TFE factor 260; repetition time (ms)/echo time (ms), 8.4/4.7; number of signals acquired, one; and acquisition time, 4 min and 56 s.

The parameters of SWI were as follows: field of view, 230 mm; matrix, 384 × 300 (768 × 768 after reconstruction); in-plane resolution, 0.30 mm × 0.30 mm; section thickness, 2 mm with overcontiguous slice; repetition time (ms)/echo time (ms), 31/7.2; echo spacing (ms), 6.2; number of echoes, four; number of signals acquired, one; flip angle, 17°; and acquisition time, 4 min and 52 s.

The parameters of DWI were as follows: field of view, 220 mm; matrix, 112 × 168 (224 × 224 after reconstruction); in-plane resolution, 0.98 mm × 0.98 mm; section thickness, 3 mm; repetition time (ms)/echo time (ms), 5,800 (shortest)/87; *b*-value, 1,000 s/mm^2^; number of signals acquired, one; and acquisition time, 1 min and 10 s.

CMBs and cortical superficial siderosis, white matter hyperintensity (WMH) and lacunar infarcts, and acute microinfarctions were evaluated using SWI, 3D-FLAIR, and DWI, respectively (Charidimou et al., 2017). Periventricular and deep WMHs were assessed according to the Fazekas rating scale (Fazekas et al., 1987). MRI images were thoroughly analyzed by two trained neurologists (Y.H. and N.K.) who were blinded to the clinical data.

### Statistical Analysis

For the analyses of the differences in demographic characteristics, the Mann-Whitney *U*-test and the χ-square test were used. To compare the difference in medical history between the group with newly detected *de novo* CMBs (positive group) and the group without *de novo* CMBs (negative group), the χ-square test was used. To compare the difference in DWI-positive lesions and Fazekas scores between the two groups, the Mann-Whitney *U*-test was used. Statistical analyses were performed using the Statistical Package for the Social Sciences Statistics software version 27 (IBM Corporation, Armonk, NY, United States).

## Results

### Patient Characteristics

**FIGURE 1:**
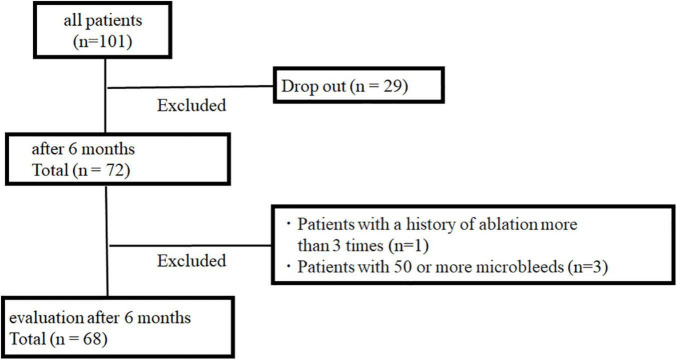
Study protocol.

A total of 101 patients underwent MRI 1–3 days after ablation. Six months after ablation, 72 patients underwent MRI, and 29 patients did not. Because of predetermined disqualification, four patients were excluded (Figure 1): one with a history of ablation more than three times, and three with more than 50 CMBs.

The baseline characteristics of the patients are presented in Table 1. The median age at baseline was 69.0 ± 9.5 (range 32–86) years, and 49 patients were male (72.1%). All patients were administered oral anticoagulants. Moreover, 45 patients had hypertension (66.2%), nine had diabetes mellitus (13.2%), 28 had dyslipidemia (41.2%), and one had a history of stroke (1.5%). None of the patients had any neurological abnormality after ablation.

**TABLE 1:** Characteristics of the 68 patients with AF.

		All	*De novo* CMB-positive group (*n* = 35)	*De novo* CMB-negative group (*n* = 33)	*p*-value
Mean age (years)		69.0 ± 9.5	67.7 ± 11.2	69.1 ± 7.4	0.980
Male sex, (n,%)		49 (72.1%)	25 (71.4%)	24 (72.7%)	0.560
Height (cm)		164.1 ± 9.2	162.8 ± 9.0	165.4 ± 9.4	0.269
Weight (kg)		65.8 ± 15.0	65.9 ± 17.5	65.7 ± 12.2	0.645
Body mass index (kg/m^2^)		24.3 ± 4.6	24.3 ± 5.1	24.0 ± 4.0	0.754
Current smoking (n,%)		34 (50.0%)	14 (40.0%)	20 (60.6%)	0.072
**Blood pressure and Heart rate**					
	Systolic blood pressure (mmHg)	132.8 ± 19.5	130.9 ± 20.2	134.8 ± 18.8	0.348
	Diastolic blood pressure (mmHg)	77.9 ± 12.8	77.8 ± 11.9	77.9 ± 13.7	0.839
	Heart rate (beat per minute)	71.9 ± 13.9	70.6 ± 13.9	73.4 ± 13.9	0.418
**Past history**					
	Hypertension (n,%)	45 (66.2%)	22 (62.9%)	23 (69.7%)	0.368
	Dyslipidemia (n,%)	28 (41.2%)	13 (37.1%)	15 (45.5%)	0.327
	Diabetes mellitus (n,%)	9 (13.2%)	5 (14.2%)	4 (12.1%)	0.539
	History of TIA and/or stroke (n,%)	1 (1.5%)	1 (2.8%)	0 (0%)	0.515
**Medication**					
	Antiplatelet (n,%)	9 (13.2%)	6 (17.1%)	3 (9.1%)	0.269
	Statin (n,%)	24 (35.2%)	12 (34.2%)	12 (36.4%)	0.529

*AF, atrial fibrillation; CMBs, cerebral microbleeds.*

### Brain Magnetic Resonance Imaging Findings 6 Months After Ablation

**FIGURE 2:**
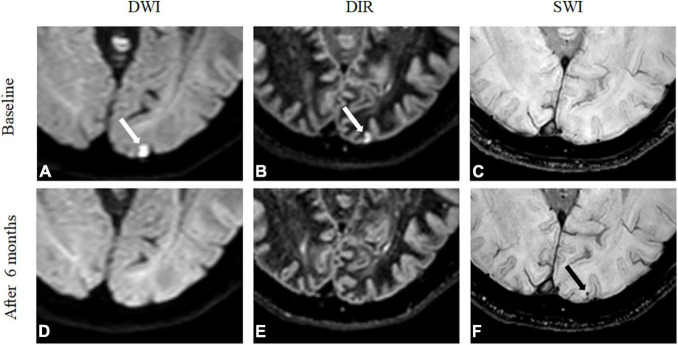
*De novo* cerebral microbleeds matched with microinfarcts. A 71-year-old woman showed an embolic microinfarct that was detected in the left occipital lobe on DWI **(A)** and 3D-DIR **(B)** at baseline, but no abnormality was found on SWI **(C)**. After 6 months, DWI **(D)** and 3D-DIR **(E)** did not detect an abnormality. A *de novo* CMB was detected in the left occipital lobe by SWI **(F)**, and the lesion was in the same location as where the embolic infarction was detected on baseline MRI.

**FIGURE 3:**
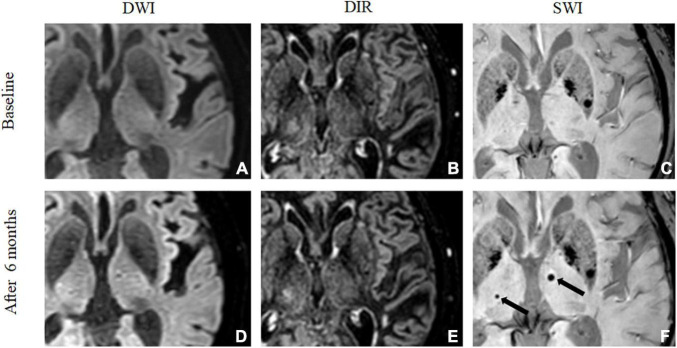
*De novo* cerebral microbleeds mismatched with microinfarcts. A 73-year-old man showed no embolic microinfarct on DWI **(A)** or 3D-DIR **(B)**, and no CMBs were found on SWI **(C)** at baseline. After 6 months, DWI **(D)** and 3D-DIR **(E)** did not detect any abnormalities. Two *de novo* CMBs were detected in the bilateral thalamus by SWI **(F)** after 6 months, but the CMBs were not related to the microinfarction.

Figures 2, 3 show two representative patterns of MRI findings. At baseline, DWI detected embolic microinfarcts in 59 out of 68 patients (86.8%), with a total of 392 lesions. These embolic microinfarcts were classified as the lobar (346), deep (11), and infratentorial lesions (35). CMBs were observed in 59 out of 68 patients (86.8%), with a total of 137 lesions on SWI at baseline. These CMBs were in the lobar (96), deep (19), and infratentorial areas (22). Six months later, MRI detected 208 CMBs with SWI. These were in the lobar (156), deep (24), and infratentorial (28) areas. Consequently, 71 *de novo* CMBs were revealed. They were in the lobar (60), deep (5), and infratentorial (6) areas. Fifty-six out of 392 (14.3%) microinfarctions transformed into CMBs. When focused on cortical microinfarctions, 51 out of 346 (14.7%) became lobar CMBs. Additionally, 56 out of 71 *de novo* CMBs (78.9%) were located in the same position where embolic microinfarctions were found in the baseline MRI (Figure 2 and Table 2).

**TABLE 2:** Number of embolic microinfarctions and CMBs at baseline and after 6 months.

	Total	Lobar	Deep	Infratentorial
Embolic microinfarctions (baseline)	392	346 (88.3%)	11 (2.8%)	35 (8.9%)
CMBs (baseline)	137	96 (70.1%)	19 (13.9%)	22 (16.1%)
CMBs (after 6 months)	208	156 (75.0%)	24 (11.5%)	28 (13.5%)
*De novo* CMBs	71	60 (84.5%)	5 (7.0%)	6 (8.5%)
CMBs due to microinfarct	56	51 (91.1%)	1 (1.8%)	4 (7.1%)
CMBs without relation to microinfarct	15	9 (60.0%)	4 (26.7%)	2 (13.3%)

*AF, atrial fibrillation; CMBs, cerebral microbleeds; DWMH, deep white matter hyperintensity; PVH, periventricular hyperintensity.*

Imaging and laboratory characteristics of patients between the *de novo* CMB-positive and *de novo* CMB-negative groups are also shown in Table 3. When we compared the positive and negative groups for *de novo* CMBs, DWI-positive lesions at baseline, especially those in the lobar area, were significantly higher in the positive group (*P* = 0.01). Furthermore, the Fazekas scores for both periventricular hyperintensity and deep white matter hyperintensity were significantly higher in the positive group than in the negative group (*P* < 0.05). Moreover, hemoglobin and hematocrit levels were significantly higher in the positive group than in the negative group (*P* < 0.05), and platelet count was significantly lower in the positive group than in the negative group (*P* < 0.01).

**TABLE 3:** MRI findings, laboratory, and echocardiography findings in the 68 patients with AF.

		All	*De novo* CMB-positive group (*n* = 35)	*De novo* CMB-negative group (*n* = 33)	*p*-value
**MRI findings**					
	**Embolic microinfarctions(baseline)**				
	Total	5.8 ± 6.9	8.1 ± 8.1	3.2 ± 4.4	0.01*
	Lobar	5.1 ± 6.2	7.0 ± 7.2	3.0 ± 4.3	0.01*
	Deep	0.2 ± 0.5	0.3 ± 0.6	0.1 ± 0.2	0.089
	Infratentorial	0.5 ± 0.9	0.9 ± 1.1	0.2 ± 0.4	0.01*
	PVH(Fazekas grade)	1.1 ± 0.9	1.3 ± 0.8	0.9 ± 0.8	0.024*
	DWMH (Fazekas grade)	1.8 ± 0.9	2.1 ± 0.87	1.6 ± 0.9	0.041*
	CMBs (baseline)				
	Total	2.0 ± 2.3	1.9 ± 1.9	2.2 ± 2.7	0.96
	Lobar	1.4 ± 1.7	1.4 ± 1.6	1.4 ± 1.8	0.63
	Deep	0.3 ± 0.7	0.2 ± 0.5	0.3 ± 0.9	0.91
	Infratentorial	0.3 ± 0.7	0.2 ± 0.6	0.4 ± 0.7	0.21
	6 months				
	Total	3.1 ± 2.7	3.9 ± 2.4	2.2 ± 2.7	0.01*
	Lobar	2.3 ± 2.1	3.1 ± 2.1	1.4 ± 1.8	0.01*
	Deep	0.4 ± 0.9	0.4 ± 0.8	0.3 ± 0.9	0.49
	Infratentorial	0.4 ± 0.7	0.4 ± 0.7	0.4 ± 0.7	0.92
	Cortical superficial siderosis (n)	1 (1.0%)	1 (0.3%)	0	0.52
	Lacune	0.9 ± 2.0	0.8 ± 2.2	1.0 ± 1.9	0.60
	Enlargement of perivascular spaces	51 (75.0%)	28 (80.0%)	23 (70.0%)	0.24
	Centrum semiovale	12 (17.6%)	7 (20.0%)	5 (15.2%)	0.42
	Basal ganglia	48 (70.6%)	26 (74.3%)	22 (66.7%)	0.34
**Laboratory data**					
	WBC (× 10^3^/μL)	5.6 ± 1.7	5.4 ± 1.6	5.9 ± 1.7	0.141
	RBC(× 10^3^/μL)	445.0 ± 56.7	454.8 ± 63.5	434.6 ± 47.3	0.071
	Hemoglobin (g/dL)	13.7 ± 1.6	14.1 ± 1.7	13.3 ± 1.4	0.026*
	Hematocrit (%)	41.2 ± 4.6	42.4 ± 4.9	39.9 ± 4.1	0.023*
	Platelet (× 103/μL)	24.1 ± 18.2	19.8 ± 4.9	28.6 ± 25.1	0.003*
	APTT(second)	36.9 ± 8.2	37.1 ± 7.0	36.8 ± 9.5	0.338
	PT (%)	14.4 ± 3.2	14.8 ± 3.8	13.9 ± 2.5	0.397
	PT-INR	1.2 ± 0.3	1.3 ± 0.4	1.2 ± 0.2	0.523
	Fibrinogen (mg/dL)	271 ± 63.0	269.0 ± 63.6	274.3 ± 63.2	0.827
	D-dimer (μg/mL)	0.6 ± 0.2	0.5 ± 0.01	0.62 ± 0.31	0.201
	BNP (ng/mL)	82.8 ± 67.4	94.8 ± 70.5	70.0 ± 62.6	0.103
**Echocardiography**					
	SV (mL)	67.8 ± 16.7	66.0 ± 16.1	69.6 ± 17.4	0.414
	LAD (mm)	41.4 ± 7.1	42.2 ± 7.6	40.6 ± 6.4	0.601
	LAVI (mL/m^2^)	46.3 ± 17.7	47.9 ± 17.7	44.7 ± 18.0	0.318
	E/e’	11.4 ± 6.3	11.7 ± 8.1	11.0 ± 3.4	0.486
	CHADS_2_ score	1.2 ± 1.0	1.2 ± 1.2	1.2 ± 0.8	0.555
	HAS-BLED	1.2 ± 0.7	1.2 ± 0.6	1.2 ± 0.8	0.826

AF, atrial fibrillation; CMBs, cerebral microbleeds; DWMH, deep white matter hyperintensity; PVH, periventricular hyperintensity; WBC, white blood cell; RBC, red blood cell; APTT, activated partial thromboplastin time; PT, prothrombin time; PT-INR, prothrombin time-international normalized ratio; BNP, brain natriuretic peptide; SV, stroke volume; LAD, left atrial dimension; LAVI, left atrial volume index. *p < 0.05 for De novo CMB positive group versus negative group.

## Discussion

In this study, we reported *de novo* CMBs in patients with AF after ablation therapy. First, *de novo* CMBs appeared in 35 (51.5%) patients 6 months after ablation therapy, and the location of almost 80% of *de novo* CMBs matched with the embolic microinfarctions detected on baseline MRI. Second, 84.5% of *de novo* CMBs were detected as the lobar type. Third, when comparing the positive and negative groups for *de novo* CMBs, the Fazekas score, and hemoglobin and hematocrit levels were higher in the positive group than in the negative group, while platelet count was significantly lower in the positive group than in the negative group.

This study revealed that *de novo* CMBs appeared after catheter ablation therapy, and most *de novo* CMBs were located spatially in correspondence to preceding embolic microinfarctions. Previous studies have reported a higher incidence of CMBs in patients with AF than in those without AF, and CMBs with AF were predominantly of the lobar type (Horstmann et al., 2015; Selim and Diener, 2017; Herm et al., 2019). Although anticoagulation therapy might be associated with the occurrence of CMBs (Werring et al., 2004; Herm et al., 2019), the detailed pathogenesis of CMBs in patients with AF remains unclear. This study revealed a novel mechanism in that some *de novo* CMBs after catheter ablation were derived from embolic microinfarctions found at baseline MRI. Our previous study showed that small cortical infarctions could cause lobar CMBs, such as hemorrhagic transformation in ischemic stroke (Ito et al., 2019). In accordance with these observation, a previous review has classified CMBs into either primary or secondary microbleeds (Fisher, 2014). Primary CMBs can be caused by the perivascular accumulation of hemosiderin-laden macrophages as a corollary of extravasated erythrocytes. Secondary CMBs may be caused by hemorrhagic transformation of microinfarction. Although our study evaluated only patients with AF after catheter ablation therapy, the present results may suggest that embolic CMBs could develop from incidentally occurring microembolism and explain why CMBs in patients with AF are predominantly of the lobar type. Moreover, when the clinician finds lobar type microbleeds, the possibility of embolic stroke due to AF should be considered besides CAA.

In the present study, the Fazekas score was higher in the positive group than in the negative group. Both periventricular and deep subcortical WMHs are often found on MRI in elderly people, and are a representative finding of cerebral small vessel disease (Wardlaw et al., 2013). Periventricular and deep WMH are closely associated with CMBs (Yamada et al., 2012). Moreover, another study has reported that CMBs are associated with recurrent stroke (Chatzikonstantinou et al., 2011). Laboratory data in the present study revealed elevated hemoglobin and hematocrit values, and a decreased platelet count in the positive group compared to the negative group. A higher viscosity and fragility of hemostasis might be associated with microvascular disturbance, leading to development of CMBs.

Several studies have reported an association between AF and dementia (Ott et al., 1997; Bunch et al., 2010; Kwok et al., 2011). Although stroke events can increase the incidence of dementia, stroke-free patients with AF also have a higher risk of cognitive impairment and dementia (Kwok et al., 2011; Madhavan et al., 2018). The existence of more than three CMBs is reportedly associated with dementia (Ding et al., 2017), and, in reality, lobar CMBs are often observed in patients with AF (Vernooij et al., 2009; Selim and Diener, 2017). Therefore, cognitive dysfunction in AF may be partially attributable to an increase in lobar CMBs. These CMBs may impair the functions of the cerebral cortex and cerebral white matter (Werring et al., 2004), since CMBs and cerebral microinfarcts are reportedly associated with cognitive dysfunction (van Veluw et al., 2015). Recent studies have shown that treatment for AF, such as anticoagulants and ablation, can prevent cognitive decline (Bunch et al., 2011; Jacobs et al., 2016; Friberg and Rosenqvist, 2018; Kim et al., 2020).

This study has some limitations. First, an MRI examination could not be performed before the ablation. Therefore, it cannot be denied that the CMBs at baseline MRI might have been affected by ablation therapy. In addition, we evaluated follow-up MRI 6 months after ablation. A previous report showed that the new CMBs can develop rapidly after acute ischemic stroke (Jeon et al., 2009), and there is a possibility that hemorrhagic transformation of the infarction could be detected at an earlier time. The other mechanism such as angiophagy (Ito et al., 2019) might be explained for occurring the *de novo* CMBs. Second, we could not compare the MRI findings of patients who underwent ablation therapy and those who did not. Because we did not enroll AF patients without ablation as a control group, the incidence of CMBs in patients with AF in our study remains unclear. Moreover, we did not follow up the MRI images of patients with AF without ablation; it was not clear whether most patients with AF also had CMBs due to microinfarcts. A prospective study comparing patients with AF with and without ablation can resolve this problem. Therefore, further studies are required.

## Conclusion

Our study showed that most *de novo* CMBs were derived from embolic microinfarctions and were of the lobar type after catheter ablation therapy in patients with AF. Some of the CMBs in patients with AF may have originated from incidental cerebral microinfarction.

## Data Availability Statement

The raw data supporting the conclusions of this article are available from the corresponding author, AS (a-shindo@med.mie-u.ac.jp), upon reasonable request.

## Ethics Statement

The studies involving human participants were reviewed and approved by the Ethical Review Board of Mie University Hospital. The patients/participants provided their written informed consent to participate in this study. Written informed consent was obtained from the individual(s) for the publication of any potentially identifiable images or data included in this article.

## Author Contributions

YH: draft of manuscript, acquisition of data, and analysis. NK, NN, KMu, AS, KMa, YI, and MSa: revision of manuscript, interpretation of data, and study supervision. K-iT and MSh: revision of manuscript and interpretation of data. TF, YK, SF, RK, MU, MM, HS, and KD: acquisition of data and interpretation of data. HT: revision of the manuscript, study concept and design, and study supervision. All authors contributed to the article and approved the submitted version.

## Conflict of Interest

The authors declare that the research was conducted in the absence of any commercial or financial relationships that could be construed as a potential conflict of interest.

## Publisher’s Note

All claims expressed in this article are solely those of the authors and do not necessarily represent those of their affiliated organizations, or those of the publisher, the editors and the reviewers. Any product that may be evaluated in this article, or claim that may be made by its manufacturer, is not guaranteed or endorsed by the publisher.

## Abbreviations

AF: atrial fibrillation
CMBs: cerebral microbleeds
MRI: Magnetic Resonance Imaging.

## References

[B1] Bunch T. J. (2020). Atrial fibrillation and dementia. *Circulation* 142 618–620. 10.1161/circulationaha.120.045866 32804567

[B2] Bunch T. J. Crandall B. G. Weiss J. P. May H. T. Bair T. L. Osborn J. S. (2011). Patients treated with catheter ablation for atrial fibrillation have long-term rates of death, stroke, and dementia similar to patients without atrial fibrillation. *J. Cardiovasc. Electrophysiol*. 22 839–845. 10.1111/j.1540-8167.2011.02035.x 21410581

[B3] Bunch T. J. Weiss J. P. Crandall B. G. May H. T. Bair T. L. Osborn J. S. (2010). Atrial fibrillation is independently associated with senile, vascular, and Alzheimer’s dementia. *Heart Rhythm*. 7 433–437. 20122875 10.1016/j.hrthm.2009.12.004

[B4] Charidimou A. Boulouis G. Gurol M. E. Ayata C. Bacskai B. J. Frosch M. P. (2017). Emerging concepts in sporadic cerebral amyloid angiopathy. *Brain* 140 1829–1850. 10.1093/brain/awx047 28334869 PMC6059159

[B5] Chatzikonstantinou A. Willmann O. Szabo K. Hennerici M. G. (2011). Cerebral microbleeds are uncommon in ischemic stroke associated with nonvalvular atrial fibrillation. *J. Neuroimaging*. 21 103–107. 10.1111/j.1552-6569.2009.00440.x 19888932

[B6] Ding J. Sigurðsson S. Jónsson P. V. Eiriksdottir G. Meirelles O. Kjartansson O. (2017). Space and location of cerebral microbleeds, cognitive decline, and dementia in the community. *Neurology* 88 2089–2097. 10.1212/WNL.0000000000003983 28468844 PMC5447401

[B7] Fazekas F. Chawluk J. B. Alavi A. Hurtig H. I. Zimmerman R. A. (1987). MR signal abnormalities at 1.5 T in Alzheimer’s dementia and normal aging. *Am. J. Roentgenol*. 149 351–356. 3496763 10.2214/ajr.149.2.351

[B8] Fisher M. (2014). Cerebral microbleeds: where are we now? *Neurology* 83 1304–1305. 10.1212/WNL.0000000000000871 25186856

[B9] Friberg L. Rosenqvist M. (2018). Less dementia with oral anticoagulation in atrial fibrillation. *Eur. Heart J*. 39 453–460. 29077849 10.1093/eurheartj/ehx579

[B10] Graff-Radford J. Madhavan M. Vemuri P. Rabinstein A. A. Cha R. H. Mielke M. M. (2016). Atrial fibrillation, cognitive impairment, and neuroimaging. *Alzheimers Dement* 12 391–398. 26607820 10.1016/j.jalz.2015.08.164 PMC4841716

[B11] Greenberg S. M. Vernooij M. W. Cordonnier C. Viswanathan A. Al-Shahi Salman R. Warach S. (2009). Cerebral microbleeds: a guide to detection and interpretation. *Lancet Neurol*. 8 165–174. 10.1016/S1474-4422(09)70013-4 19161908 PMC3414436

[B12] Herm J. Schurig J. Martinek M. R. Höltgen R. Schirdewan A. Kirchhof P. (2019). MRI-detected brain lesions in AF patients without further stroke risk factors undergoing ablation - a retrospective analysis of prospective studies. *BMC Cardiovasc. Disord*. 19:58. 10.1186/s12872-019-1035-1 30871479 PMC6419420

[B13] Horstmann S. Mohlenbruch M. Wegele C. Rizos T. Laible M. Rauch G. (2015). Prevalence of atrial fibrillation and association of previous antithrombotic treatment in patients with cerebral microbleeds. *Eur. J. Neurol*. 22 1355–1362. 10.1111/ene.12608 25557113

[B14] Ii Y. Maeda M. Ishikawa H. Ito A. Matsuo K. Umino M. (2019). Cortical microinfarcts in patients with multiple lobar microbleeds on 3 T MRI. *J. Neurol.* 266 1887–1896. 10.1007/s00415-019-09350-9 31049727

[B15] Ii Y. Maeda M. Kida H. Matsuo K. Shindo A. Taniguchi A. (2013). *In vivo* detection of cortical microinfarcts on ultrahigh-field MRI. *J. Neuroimaging*. 23 28–32. 10.1111/j.1552-6569.2012.00722.x 22607584

[B16] Ito A. O. Shindo A. Ii Y. Ishikawa H. Taniguchi A. Shiba M. (2019). Small cortical infarcts transformed to lobar cerebral microbleeds: a case series. *J. Stroke Cerebrovasc. Dis*. 28 e30–e32. 10.1016/j.jstrokecerebrovasdis.2018.12.050 30655044

[B17] Jacobs V. May H. T. Bair T. L. Crandall B. G. Cutler M. J. Day J. D. (2016). Long-Term population-based cerebral ischemic event and cognitive outcomes of direct oral anticoagulants compared with warfarin among long-term anticoagulated patients for atrial fibrillation. *Am. J. Cardiol*. 118 210–214. 10.1016/j.amjcard.2016.04.039 27236255

[B18] Jeon S. B. Kwon S. U. Cho A. H. Yun S. C. Kim J. S. Kang D. W. (2009). Rapid appearance of new cerebral microbleeds after acute ischemic stroke. *Neurology* 73 1638–1644. 10.1212/WNL.0b013e3181bd110f 19759365

[B19] Kato N. Muraga K. Hirata Y. Shindo A. Matsuura K. Ii Y. (2021). Brain magnetic resonance imaging and cognitive alterations after ablation in patients with atrial fibrillation. *Sci. Rep*. 11:18995. 10.1038/s41598-021-98484-w 34556757 PMC8460624

[B20] Kim D. Yang P. S. Sung J. H. Jang E. Yu H. T. Kim T. H. (2020). Less dementia after catheter ablation for atrial fibrillation: a nationwide cohort study. *Eur. Heart J*. 41 4483–4493. 10.1093/eurheartj/ehaa726 33022705

[B21] Kwok C. S. Loke Y. K. Hale R. Potter J. F. Myint P. K. (2011). Atrial fibrillation and incidence of dementia: a systematic review and meta-analysis. *Neurology* 76 914–922. 10.1212/wnl.0b013e31820f2e38 21383328

[B22] Madhavan M. Graff-Radford J. Piccini J. P. Gersh B. J. (2018). Cognitive dysfunction in atrial fibrillation. *Nat. Rev. Cardiol*. 15 744–756. 30275499 10.1038/s41569-018-0075-z

[B23] Matsuyama H. Ii Y. Maeda M. Umino M. Ueda Y. Tabei K. I. (2017). Background and distribution of lobar microbleeds in cognitive dysfunction. *Brain Behav*. 7:e00856. 10.1002/brb3.856 29201555 PMC5698872

[B24] Ogawa Ito A. Shindo A. Ii Y. Matsuura K. Tabei K. I. Maeda M. (2019). Microbleeds after carotid artery stenting: small embolism may induce cerebral microbleeds. *Cerebrovasc. Dis. Extra* 9 57–65. 10.1159/000500112 31203282 PMC6600049

[B25] Ott A. Breteler M. M. de Bruyne M. C. van Harskamp F. Grobbee D. E. Hofman A. (1997). Atrial fibrillation and dementia in a population-based study: the Rotterdam study. *Stroke* 28 316–321. 10.1161/01.str.28.2.316 9040682

[B26] Poels M. M. Ikram M. A. van der Lugt A. Hofman A. Niessen W. J. Krestin G. P. (2012). Cerebral microbleeds are associated with worse cognitive function: the Rotterdam scan study. *Neurology* 78 326–333. 10.1212/WNL.0b013e3182452928 22262748

[B27] Saito T. Kawamura Y. Tanabe Y. Asanome A. Takahashi K. Sawada J. (2014). Cerebral microbleeds and asymptomatic cerebral infarctions in patients with atrial fibrillation. *J. Stroke Cerebrovasc. Dis*. 23 1616–1622. 10.1016/j.jstrokecerebrovasdis.2014.01.005 24680089

[B28] Selim M. Diener H. C. (2017). Atrial fibrillation and microbleeds. *Stroke* 48 2660–2664. 28916675 10.1161/STROKEAHA.117.017085

[B29] Sutter J. S. Lokhnygina Y. Daubert J. P. Bahnson T. Jackson K. Koontz J. I. (2020). Safety and efficacy outcomes of left atrial posterior wall isolation compared to pulmonary vein isolation and pulmonary vein isolation with linear ablation for the treatment of persistent atrial fibrillation. *Am. Heart J*. 220 89–96. 10.1016/j.ahj.2019.11.010 31805423

[B30] van Veluw S. J. Hilal S. Kuijf H. J. Ikram M. K. Xin X. Yeow T. B. (2015). Cortical microinfarcts on 3T MRI: clinical correlates in memory-clinic patients. *Alzheimers Dement*. 11 1500–1509. 10.1016/j.jalz.2014.12.010 25956990

[B31] Vernooij M. W. Haag M. D. van der Lugt A. Hofman A. Krestin G. P. Stricker B. H. (2009). Use of antithrombotic drugs and the presence of cerebral microbleeds: the Rotterdam Scan Study. *Arch. Neurol*. 66 714–720. 10.1001/archneurol.2009.42 19364926

[B32] Wardlaw J. M. Smith E. E. Biessels G. J. Cordonnier C. Fazekas F. Frayne R. (2013). Neuroimaging standards for research into small vessel disease and its contribution to ageing and neurodegeneration. *Lancet Neurol*. 12 822–838. 10.1016/S1474-4422(13)70124-8 23867200 PMC3714437

[B33] Werring D. J. Frazer D. W. Coward L. J. Losseff N. A. Watt H. Cipolotti L. (2004). Cognitive dysfunction in patients with cerebral microbleeds on T2*-weighted gradient-echo MRI. *Brain* 127 2265–2275. 10.1093/brain/awh253 15282216

[B34] Yamada S. Saiki M. Satow T. Fukuda A. Ito M. Minami S. (2012). Periventricular and deep white matter leukoaraiosis have a closer association with cerebral microbleeds than age. *Eur. J. Neurol*. 19 98–104. 10.1111/j.1468-1331.2011.03451.x 21645176

[B35] Yates P. A. Desmond P. M. Phal P. M. Steward C. Szoeke C. Salvado O. (2014). Incidence of cerebral microbleeds in preclinical Alzheimer disease. *Neurology* 82 1266–1273. 10.1212/WNL.0000000000000285 24623839 PMC4001205

